# Dyadic adjustment and parenting stress in internationally adoptive mothers and fathers: the mediating role of adult attachment dimensions

**DOI:** 10.3389/fpsyg.2015.01279

**Published:** 2015-09-02

**Authors:** Silvia Salcuni, Diana Miconi, Gianmarco Altoè, Ughetta Moscardino

**Affiliations:** Department of Developmental Psychology and Socialization, University of Padova, Padova, Italy

**Keywords:** adoptive parents, adult attachment, parenting stress, dyadic adjustment, international adoption

## Abstract

Previous research has shown that a positive marital functioning represents a resource in adoptive families, leading to a decrease in parenting stress, but little is known about the factors mediating such a relationship. This study aimed to explore whether adult attachment avoidance and anxiety mediate the effect of dyadic functioning on parenting stress in 90 internationally adoptive couples (mothers and fathers) who had adopted a child (aged 3–10 years) in the last 36 months. Participants completed self-report measures of dyadic adjustment, adult attachment, and parenting stress. A series of path analyses supported the mediation hypothesis, but differentially for mothers and fathers. Among mothers, there was a direct and negative relationship between dyadic adjustment and parenting stress. In addition, a better dyadic adjustment was related to lower levels of attachment anxiety, which in turn were associated with less parenting stress. Among fathers, increased dyadic adjustment was related to lower levels of attachment avoidance, which in turn were associated with reduced parenting stress. These findings suggest the importance of including both mothers and fathers in adoption research. Adoptive parents could benefit from specific interventions aimed at reducing attachment avoidance and anxiety by supporting parental sense of competence and involvement for mothers and fathers, respectively.

## Introduction

Parenting stress is a complex construct determined by multiple sources, including parent, child and situational factors related to parent–child interaction (Abidin, 1995) and interferes with many aspects of family functioning, such as positive parenting practices and child psychosocial adjustment (Cummings et al., 2000; Greenley et al., 2006). Parenting stress, especially during the delicate phase of transition to parenthood, has been extensively studied in its association with marital quality (Cowan and Cowan, 1995). However, most studies so far have focused on biological parents, whereas less is known about the associations between dyadic functioning and parenting stress in the context of adoptive parenthood, especially in the post-adoption period (McKay et al., 2010).

Adoption may be a detrimental factor for parenting stress and marital satisfaction, as adoptive parents face unique challenges linked to both life events (e.g., infertility, suddenly becoming parents, adoption stigma) and child characteristics (e.g., children adopted at an older age, history of adversity, behavioral and emotional problems; Glidden, 2000; Nickman et al., 2005; Goldberg, 2010). Moreover, in the context of inter-country adoptions parents have to deal with the additional stressor of adopting children who might come from a different ethnic group (Lazarus et al., 2002). Such risk factors could account for findings that report higher parenting stress in adoptive parents compared to biological parents (McGlone et al., 2002; Rijk et al., 2006). At the same time, adoptive parents present some advantages over their biological counterparts, such as being older, financially secure, with a stable career and married longer, which could help them face the additional stressors linked to the adoption process mentioned above (Brodzinsky and Huffman, 1988; Levy-Shiff et al., 1991; Salcuni et al., 2003, 2006). Another important protective factor is the quality of dyadic functioning Lionetti et al., 2015). Recent findings highlight how the presence of a solid and positive marital relationship represents a resource in adoptive families, leading to a decrease in parenting stress and to better family and child adjustment post-adoption (Ceballo et al., 2004; Goldberg et al., 2010; Goldberg and Smith, 2014). Lionetti et al. (2015) showed how unresolved attachment in parents predicted their level of perceived stress to a greater extent than insecure attachment, together with low parenting alliance. Differences between mothers and fathers were also found. These findings are in line with the most recent trends in adoption research, which highlight the importance of family and parenting processes as predictors of child and parent outcomes (Palacios and Brodzinsky, 2010; Grotevant and McDermott, 2014), viewed as important points of entry for prevention and intervention efforts (Goldberg and Smith, 2014; Lionetti et al., 2015).

Parents’ adult attachment dimensions have been extensively linked to both marital satisfaction and parent–child adjustment in adoptive and biological families (Erel and Burman, 1995; Roberson, 2006; Mikulincer and Shaver, 2007). Attachment dimensions refer to aspects of avoidance and anxiety in establishing interpersonal relationships. Avoidance is characterized by discomfort with intimacy and dependency in relationships, whereas anxiety reflects fears of abandonment and rejection together with a strong desire for closeness in relationships (Shaver and Mikulincer, 2002). Increasingly in the literature, adult attachment is considered to be responsive to environmental circumstances, especially to the quality of ongoing relationships (Bowlby, 1973; Cozzarelli et al., 2003; Moreira et al., 2003; Simpson et al., 2003), and the transition to adoptive parenthood can clearly be considered as a major life event able to activate and change parents’ attachment systems (Bowlby, 1973; Jones et al., 2015). Moreover, recent findings show that dimensional measures, rather than categorical ones, provide a better conceptualization of adult attachment (Roisman et al., 2007; Jones et al., 2015), as they can help to explain how anxiety and avoidance independently relate to parenting. In line with these results, Green et al. (2007) found that attachment anxiety was a mediator in the relationship between social support and parenting outcomes in a sample of low SES, at risk mothers. Specifically, increased social support was linked to lower levels of attachment anxiety which, in turn, were related to better parent–child activities. However, the extent to which these results may apply to adoptive parents remains unclear.

Most studies so far have found that avoidance and anxiety are related to greater parenting stress in both mothers and fathers (Jones et al., 2015). Overall, the literature on gender issues in the attachment field shows that men report higher avoidance and lower anxiety compared to women (Del Giudice, 2011). However, findings are still inconsistent as regards the role of attachment dimensions and parent gender in the experience of parenting stress. In some cases, avoidance has been found to negatively influence parenting stress, especially for mothers (Rholes et al., 2006), whereas other research reports that anxiety is the best predictor of parenting stress both for mothers and fathers (Nygren et al., 2012). These contrasting results may be due to the heterogeneity of samples and measures used in prior studies, and highlight the need to include both parents and the use of dimensional measures to study the role of parent gender and adult attachment dimensions in parenting research (Jones et al., 2015).

The current study aims to investigate the relationships between dyadic adjustment, attachment dimensions and parenting stress among mothers and fathers of children internationally adopted in the past 36 months. Although both attachment dimensions and the quality of dyadic functioning have been shown to impact on parenting stress, little research has examined how these variables are associated with parenting outcomes (Green et al., 2007; Jones et al., 2015) and these relationships remain virtually unexplored among adoptive families. Based on the extant literature, it was hypothesized that (1) better dyadic functioning would be related to lower levels of attachment avoidance, attachment anxiety, and parenting stress; (2) attachment avoidance and anxiety would be positively associated with parenting stress, and (3) adult attachment dimensions would mediate the relationship between dyadic adjustment and parenting stress. Specifically, we expected a better dyadic adjustment to be linked to lower levels of both attachment avoidance and anxiety, which, in turn, would be related to lower levels of parenting stress (Moreira et al., 2003; Green et al., 2007; Goldberg and Smith, 2014; Jones et al., 2015). Due to existing evidence of gender differences in adult attachment (Del Giudice, 2011), it was expected that fathers would be more likely to report higher avoidance in attachment compared to mothers. Hence, we also examined whether the mediational model would differ between mothers and fathers. Given the lack of research investigating the links between dyadic adjustment, adult attachment, and parenting stress in adoptive fathers, no *a priori* hypothesis was formulated in this regard. In our study, we also included parent age, child age at assessment, child gender, number of adopted children, length of time the child spent in the adoptive family and child behavioral and emotional problems as control variables, because these factors have been previously linked to parenting stress. Specifically, a younger parental age (Mainemer et al., 1998), school-aged children (Palacios and Sánchez-Sandoval, 2005), a shorter time spent by the child in the adoptive family (Goldberg and Smith, 2014), more child emotional and behavioral problems reported by parents (Smith et al., 2001; Farr et al., 2010), as well as adopting a boy (Palacios and Sánchez-Sandoval, 2006) or more than one child (Bird et al., 2002), all represent potential risk factors for elevated levels of parenting stress.

## Materials and Methods

### Participants

Participants included *n* = 90 mother-father pairs who adopted *n* = 90 children (*n* boys = 51; 57%) through international adoption. Participants all lived in Northern Italy. Inclusion criteria were: (a) married couples; (b) children were from intact families (i.e., both the mother and father lived at home and both participated in the study); (c) no reported parents’ psychiatric illness; (d) parents did not have any biological children; (e) participants adopted a child via international adoption in the last 3 years. The average length of marriage was 12.60 years (SD = 4.84). Mothers were on average 43.44 years old (SD = 4.44), while fathers were 45.28 years old (SD = 4.58). According to Hollingshead’s index (1975), the vast majority of adoptive parents (74%) were well educated and middle to upper-middle class. Most families adopted one single child (81%), while 19% adopted two or more children. To achieve independence, we randomly selected one child from families with more than one adopted child in the last 36 months. The majority of children included in the current sample came from Eastern Europe (40%), followed by Latin American countries (33%), and by Asian countries (19%). Only 8% of children came from Africa. At the time of adoption, children ranged in age from 9 months to 10 years, with a mean of 4.98 years (SD = 2.41). At the time of the study, children were 3 to 10 years old (*M* = 6.52, SD = 2.36) and had been residing in their adoptive homes on average for 18.47 months (SD = 12.05, ranging from 1 to 36 months). The demographic characteristics reported in our sample are in line with Italian adoptive couples’ socio-demographic characteristics (Official data provided by the Government Central Authority, www.commissioneadozioni.it).

### Procedure

This study was conducted in compliance with the ethical standards for research outlined in the Ethical Principles of Psychologists and Code of Conduct (American Psychological Association, 2010). Approval from the Ethical Committee for Psychological Research of the University of Padua was obtained (Protein Number 1213/2012). Adoptive families were recruited through agencies working in the field of international adoption in Northern Italy and asked to take part in a research project on family adaptation in the post-adoption period. All parents who agreed to participate signed their informed consent and completed a set of self-report questionnaires. Confidentiality was assured by replacing parents’ personal information with a numeric code. No incentives were awarded and voluntary participation was emphasized. A total of 153 adoptive couples were contacted, of whom 104 participated in this study, with a response rate of approximately 68%. Among the participants, 14 couples (13.46%) were excluded from data analysis either due to missing values (*n* = 5, see paragraph 2.4 on data analysis for details about procedural aspects) or because they did not meet our inclusion criteria (*n* = 9), resulting in a final sample of 90 couples.

### Measures

#### Demographic and Control Variables

Parents’ and children’s demographic variables were collected using a questionnaire developed specifically for the current study. Information about adoptive families’ Socio-Economic Status (SES; Hollingshead, 1975) was obtained. Variables unique to adoption were surveyed, such as age of the child at adoption, length of time spent by the child in the adoptive family and number of adopted children. Parental perception of children’s emotional and behavioral problems were used as control variables and measured by the Strengths and Difficulties Questionnaire-Parent Version (SDQ-Parent Version; Goodman, 1997). SDQ is a brief behavioral screening questionnaire asking parents of children aged 2 to 16 years about 25 attributes. The 25 items are divided between five scales of five items each, generating scores for Conduct Problems, Inattention-Hyperactivity, Emotional Symptoms, Peer Problems and Prosocial Behavior; all scales but the last are summed to generate a Total Difficulties score, which were considered in the present research. Parents are asked to rate their children’s behaviors on a Likert Scale ranging from 0 (not true) to 2 (absolutely true). The Italian version of the instrument is available and has shown good psychometric properties (Marzocchi et al., 2002). Further information about reliability and convergent validity can be found on www.sdqinfo.com. In the present study, the Total Difficulties score was taken into account as a control variable in our mediation model. Cronbach’s α for this scale was α = 0.76 for mothers and α = 0.74 for fathers (for mothers *M* = 9.46, SD = 4.94, range 1–25; for fathers *M* = 9.41, SD = 4.87, range 0–21).

#### Dyadic Adjustment

The Dyadic Adjustment Scale (DAS; Spanier, 1976) is currently the most widely used self-report measure of relationship adjustment in the social and behavioral sciences. Four factors (Consensus, Satisfaction, Cohesion, Affectional Expression) load on one, higher order factor (Adjustment). The scale contains 32 Likert scale items that provide information on four different subscales: Relationship satisfaction, Positive relationship behaviors, Similarity in goals and beliefs and Affectional expression. The total score ranges from 0 to 151, where higher values indicate a general better level of marital adjustment. The Italian version of the questionnaire was validated by Gentili et al. (2002). A confirmatory factor analysis confirmed the factors of the original version and good internal reliability was found. In the present study, Cronbach’s alpha for the Total adjustment score was α = 0.86 for mothers, and α = 0.82 for fathers.

#### Parents’ Attachment Dimensions

The Experiences in Close Relationships Scale (ECR; Brennan et al., 1998) is a 36-item self-report measure of adult attachment providing a measure of both attachment-related avoidance (18 items; e.g., “I prefer not to show others how I feel deep down”) and anxiety (18 items; e.g., “I want to get very close to others, and this sometimes scares them away”) in close relationships. Participants had to indicate the extent to which they agreed with each statement on a 7-point Likert scale ranging from 1 (disagree strongly) to 7 (agree strongly). The ECR has demonstrated excellent psychometric properties including internal consistency, test–retest reliability, and construct validity (Brennan et al., 1998; Mikulincer and Shaver, 2007). The ECR Italian validation confirmed these excellent psychometric properties (α = 0.89 for both avoidance and anxiety; Picardi et al., 2002). In the present study, both attachment dimensions demonstrated high internal consistency (for avoidance, α = 0.83 for mothers and α = 0.87 for fathers; for anxiety, α = 0.84 for both mothers and fathers).

#### Parenting Stress

The Parenting Stress Index-Short Form (PSI-SF; Abidin, 1995) is a 36-item self-report questionnaire that asks parents of children on a Likert scale ranging from 1 (“strongly disagree”) to 5 (“strongly agree”) the degree to which they are experiencing stress in relation to their parenting role. The PSI-SF yields a Total Stress score and three subscales labeled according to the source of stress: Parental distress (PD), Parent–child dysfunctional interaction (P-CDI), and Difficult child (DC). The PSI-SF Total Stress score is obtained by adding all items, with possible scores ranging from 36 to 180. The Italian version of the questionnaire has shown good psychometric properties (Guarino et al., 2008). In the present study, Cronbach’s α for the Total Stress score was α = 0.92 for mothers and α = 0.91 for fathers.

### Data Analysis

Prior to conducting the analyses, exploratory statistics and graphs (i.e., boxplots) were used to assess normality and check for the presence of outliers on study variables. No relevant departure from normality assumptions and no extreme outliers were identified. Imputation of missing values for all variables was performed using the PASW Statistics, Release Version 18.0 (SPSS Inc, 2009). Cases were eliminated when 10% or more of the items of one measure did not receive an answer (Muris et al., 2006), resulting in a final sample of 90 adoptive couples. The missing values were imputed based upon values observed in other cases that had a similar response pattern over a set of matching variables. Descriptive information for the sample was summarized using means and standard deviations for continuous variables and counts and proportions for categorical variables. Differences between mothers and fathers were assessed using paired *t*-tests, interpreted on the basis of their significance at the 0.05 level and of Cohen’s *d* measure of effect-size (Cohen, 1988). Bivariate associations among study variables were assessed using Pearson’s correlations. At the multivariate level, the pattern of relationships specified by our theoretical model was examined through a series of path analyses (i.e., structural equation modeling for observed variables), using the package Lavaan (Rosseel, 2012) of the software R (R Development Core Team, 2013) and using a single observed score for each construct included in the model, separately for mothers and fathers. Data were analyzed using the maximum likelihood method with robust standard errors estimator. The mediating role of attachment anxiety and avoidance was evaluated using the Sobel test for mediation (Baron and Kenny, 1986; MacKinnon et al., 1995) with robust standard error estimate.

To evaluate the goodness of fit of the models, the *R^2^* of each endogenous variable and several other indices were considered (Schermelleh-Engel et al., 2003). Since the *χ*^2^ statistic is extremely sensitive to sample size, two relative fit indices have been considered: the non-normed fit index (NNFI) and the comparative fit index (CFI), as they both perform well with small and large samples. For these indices, values that are > 0.95 and > 0.97 are associated with acceptable and good fit, respectively (Schermelleh-Engel et al., 2003). The root mean square error of approximation (RMSEA) was also used. This is an absolute fit index that assesses the approximation of parameter estimates to true parameters in the population. RMSEA values that are < 0.05 can be considered as a good fit, whereas values between 0.05 and 0.08 are thought to be an adequate fit (Schermelleh-Engel et al., 2003).

## Results

### Preliminary Analyses

#### Means and Group Differences

Means and standard deviations for study variables appear in Table 1 separately for mothers and fathers. Fathers reported higher attachment avoidance than mothers. The remaining scales showed no gender differences.

**TABLE 1:** **Descriptive statistics of study variables for mothers (***n*** = 90) and fathers (***n*** = 90)**.

**Scale**	****	***M***	**SD**	**Range**	**Paired *t*-test (*df* = 89)**	**Cohen’s *d***
Dyadic Adjustment	Mothers	122.93	11.64	89–146	*t* = –0.38; *p* = 0.708	0.04
	Fathers	123.29	10.14	84–146		
Attachment avoidance	Mothers	32.03	12.20	18–74	*t* = –2.10; *p* = 0.038	0.22
	Fathers	34.88	13.41	18–80		
Attachment anxiety	Mothers	54.81	17.68	22–94	*t* = 1.50; *p* = 0.138	0.16
	Fathers	51.58	16.28	24–97		
Parenting stress	Mothers	69.76	17.26	37–111	*t* = –0.88; *p* = 0.381	0.09
	Fathers	71.10	16.55	40–120		

#### Correlations

Intercorrelations among study variables are reported in Table 2 separately for mothers and fathers, together with correlations between partner reports. As the matrix shows, correlations showed the expected pattern of association for both mothers and fathers. A better marital relationship was negatively related to parenting stress and to attachment avoidance and anxiety, whereas both attachment dimensions were positively related to parenting stress. Moderate correlations between partner reports on all relevant study variables were found, highlighting a moderate agreement in the perception of mothers and fathers as regards individual functioning and the perception of child difficulties.

**TABLE 2:** **Intercorrelations among study variables for mothers (***n*** = 90) and fathers (***n*** = 90) and correlations between mother and father reports**.

**Variable**	**1**	**2**	**3**	**4**
1 Dyadic adjustment	0.67***	–0.62***	–0.41***	–0.31**
2 Attachment avoidance	–0.66***	0.50***	0.54***	0.32**
3 Attachment anxiety	–0.42***	0.67***	0.28**	0.32**
4 Parenting stress	–0.33**	0.30**	0.26*	0.63***

Values above the diagonal are for mothers, values below are for fathers. Correlations between mother and father reports are shown in the diagonal. *p < 0.05; **p < 0.01; ***p < 0.001.

### Model Assessment

**FIGURE 1:**
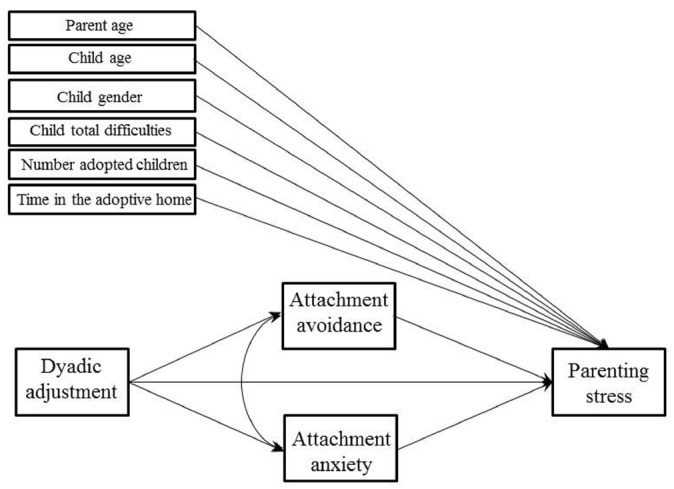
**Baseline theoretical model**.

Path analysis was used to evaluate the contributions of dyadic adjustment and attachment dimensions to parenting stress at a multivariate level. A direct relationship between dyadic adjustment and parenting stress was hypothesized, and an indirect relationship between these two variables via the mediating role of attachment avoidance and anxiety. Bivariate correlations were allowed between the two mediators. Control variables were inserted in the model as covariates on parenting stress. A graphical representation of the baseline theoretical model is presented in Figure 1. Specifically, in order to select the most plausible model that explains data (i.e., the model that represents the best compromise between fit and parsimony), we started from the baseline model and removed path coefficients based on their significance at the 5% level, their size, and in accordance with theoretical reasons. At each step, the goodness of fit of the new model (i.e., the one with less parameters) was assessed and compared with the goodness of fit of the previous model in terms of explained variance and several fit indices for structural equation models (i.e., chi-square, CFI, NNFI and RMSEA).

#### Mothers

**FIGURE 2:**
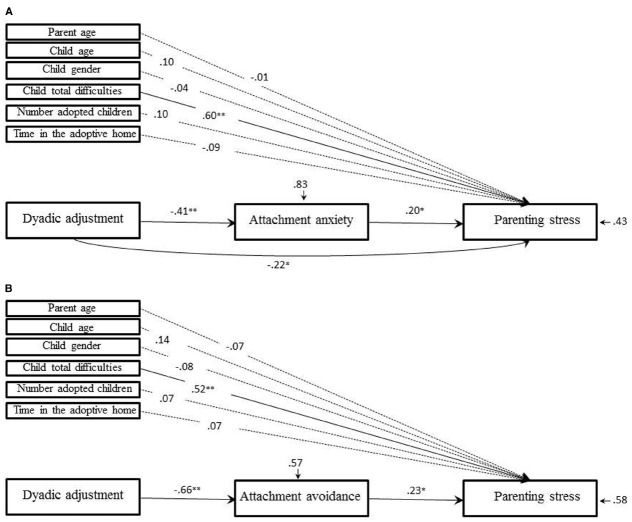
**(A)** and **(B).** Final path analytic models of the effects of dyadic adjustment and adult attachment on parenting stress in adoptive mothers **(A)** (*n* = 90) and fathers **(B)** (*n* = 90). Coefficients are STANDARDIZED structural coefficients. Dotted lines represent non-significant coefficients at the 0.05 level. *p < 0.01; **p < 0.001.

The baseline model showed a direct and negative link between dyadic adjustment and parenting stress (β = –0.30, SE = 0.13, *z* = –2.36, *p* = 0.018; β_STANDARDIZED_ = –0.21). Results of the Sobel test supported a mediating role of attachment anxiety in links between dyadic adjustment and parenting stress (β = –0.12, SE = 0.05, *z* = –2.31, *p* = 0.021; β_STANDARDIZED_ = –0.08), but did not support the mediating role of attachment avoidance (β = –0.02, SE = 0.09, *z* = –0.19, *p* = 0.852; β_STANDARDIZED_ = –0.01). The whole model accounted for 57% of the variance for parenting stress, 38% of the variance for attachment avoidance and 16% of the variance for attachment anxiety. The fit indices of the model were good (NNFI = 1.113; CFI = 1.00; RMSEA < 0.001) and the chi square was not significant [*χ*^2^ = 4.664 (12, *n* = 90), *p* = 0.968], providing a good fit to the data. Despite the good *R^2^* and fit indices, we removed attachment avoidance from the model since the link between dyadic adjustment and parenting stress via attachment avoidance was not significant at the 5% level with a small effect size. The fit indices of the model remained excellent (NNFI = 1.108; CFI = 1.00; RMSEA < 0.001), the chi square remained non-significant [*χ*^2^ = 2.56 (6, *n* = 90), *p* = 0.862], and the *R^2^* did not change, showing that the whole model accounted for 57% of the variance for parenting stress, confirming this final model as the most plausible for the observed data. Figure 2A shows the final path analytic model for mothers. In this model, dyadic adjustment was directly and negatively associated with parenting stress (β = –0.32, SE = 0.11, *z* = –2.94, *p* = 0.003; β_STANDARDIZED_ = –0.22). In addition, the Sobel test confirmed that dyadic adjustment predicted parenting stress via attachment anxiety (β = –0.123, SE = 0.05, *z* = –2.49, *p* = 0.013; β_STANDARDIZED_ = –0.08). More specifically, dyadic adjustment was negatively associated with attachment anxiety (β = –0.62, SE = 0.13, *z* = –4.66, *p* < 0.001; β_STANDARDIZED_ = –0.41), which in turn was positively associated with parenting stress (β = 0.20, SE = 0.07, *z* = –2.83, *p* = 0.005; β_STANDARDIZED_ = –0.20). Thus, among mothers the relationship of dyadic adjustment to parenting stress was partially mediated via attachment anxiety.

#### Fathers

As regards fathers, the baseline model did not show a direct link between dyadic adjustment and parenting stress (β = –0.24, SE = 0.24, *z* = –0.97, *p* = 0.331; β_STANDARDIZED_ = –0.15). Despite the good *R^2^* (44% of the variance for parenting stress, 43% of the variance for attachment avoidance and 17% of the variance for attachment anxiety), this model did not provide a good fit to the data. The fit indices of the model were not acceptable (NNFI = 0.787; CFI = 0.894, RMSEA = 0.120) and the chi-square was significant [*χ*^2^ = 27.423(12, *n* = 90), *p* = 0.007]. As a next step, since the direct link between dyadic adjustment and parenting stress was not significant at the 5% level with a small effect size, we decided to remove this link from the model. The fit indices of the model improved, but still did not provide a good fit to the data (NNFI = 0.819, CFI = 0.902, RMSEA = 0.110) and the chi-square remained significant [*χ*^2^ = 27.178 (13, *n* = 90), *p* = 0.012]. The Sobel test supported a mediating role of attachment avoidance (β = –0.30, SE = 0.13, *z* = –2.35, *p* = 0.019; β_STANDARDIZED_ = –0.18), but not of attachment anxiety (β = 0.041, SE = 0.07, *z* = 0.55, *p* = 0.580; β_STANDARDIZED_ = 0.03) in the relation between dyadic adjustment and parenting stress. Therefore, attachment anxiety was removed from the model. Figure 2B shows the final path analytic model for fathers. In this model, dyadic adjustment was negatively associated with attachment avoidance (β = –0.870, SE = 0.10, *z* = –8.35, *p* < 0.001; β_STANDARDIZED_ = –0.66), which in turn was positively associated with parenting stress (β = 0.29, SE = 0.09, *z* = 3.15, *p* = 0.002; β_STANDARDIZED_ = 0.23). Thus, among fathers the relationship of dyadic adjustment to parenting stress was fully mediated through attachment avoidance and this indirect link was confirmed by the Sobel test (β = –0.25, SE = 0.09, *z* = –2.83, *p* = 0.005; β_STANDARDIZED_ = –0.15). The fit indices improved in this final model, providing a good fit to the data (NNFI = 0.932; CFI = 0.968; RMSEA = 0.067) and the chi-square became non-significant [*χ*^2^ = 9.845 (7, *n* = 90), *p* = 0.198]. In addition, the *R^2^* remained almost unchanged compared to the baseline model, showing that the whole model accounted for 42% of the variance for parenting stress, confirming this final model as the most plausible for the observed data^1^.

## Discussion

Dyadic functioning has been shown to have a positive influence on parenting quality in both adoptive and non-adoptive families, but less is known about the factors mediating this relationship. The current study set out to investigate the role of dyadic adjustment, attachment anxiety and attachment avoidance in predicting parenting stress among mothers and fathers who had adopted a child via international adoption in the last 3 years. Path analytic models were used separately for mothers and fathers to examine whether dyadic adjustment would be related to parenting stress, and whether this link would be mediated by adult attachment dimensions (i.e., anxiety and avoidance). In addition, the focus of interest was on whether the mediational model would differ across parental gender. Results supported the mediational role of adult attachment dimensions, but differentially for mothers and fathers.

Consistent with previous findings (Leve et al., 2001; Viana and Welsh, 2010; Goldberg and Smith, 2014), this study documented the overall protective role of positive dyadic adjustment and low adult attachment anxiety and avoidance on parenting stress in adoptive families. On average, mothers and fathers had a positive perception of their relationship and reported a non-clinical level of parenting stress (Rosnati et al., 2013). In line with our hypotheses, a better dyadic adjustment was negatively related to attachment anxiety and avoidance and to parenting stress, both for mothers and fathers. In addition, both attachment dimensions (i.e., avoidance and anxiety) were positively related to parenting stress for both parents, confirming our expectations. Mothers and fathers did not differ in their overall perceived level of dyadic functioning and parenting stress; however, the inter-correlations between mother and father variables showed a moderate agreement, suggesting that, despite these similar perceptions, they nonetheless provide a somewhat different perspective on their individual, dyadic, and parental functioning (Rosnati et al., 2008). Specifically, one difference emerged as a function of parental gender. As expected, adoptive fathers reported higher attachment avoidance than mothers, in line with previous findings from attachment research (Rholes et al., 2006) showing that men are more avoidant and less anxious than women. While gender differences in anxiety peak during early adulthood and decrease over time, differences in avoidance between men and women increase later in life, supporting our findings (Del Giudice, 2011).

As anticipated, our path analytic models also differed as a function of parental gender. The hypothesized mediational role of attachment dimensions in the relationship between dyadic adjustment and parenting stress was confirmed for both mothers and fathers, but with some relevant differences. Among mothers, a better dyadic adjustment was related to lower levels of attachment anxiety, which in turn were associated with decreased parenting stress. On the contrary, increased dyadic adjustment among fathers was linked to lower levels of attachment avoidance, but not to anxiety, which in turn were associated with lower levels of parenting stress. In addition, a direct and negative relationship between dyadic adjustment and parenting stress emerged, but only among mothers. These results only partially confirm our expectations of a link between attachment avoidance and anxiety and parenting stress in both mothers and fathers (Jones et al., 2015). However, Rholes et al. (2006), in their study on a sample of married couples after the birth of their first child, found that avoidant mothers and fathers showed more parenting stress, even if this relation was stronger among women. On the other hand, Nygren et al. (2012) found attachment anxiety to be associated with more parenting stress in parents of toddlers and did not find any gender differences, although their sample consisted mostly of mothers. In line with Nygren’s results, Green et al. (2007) found anxiety, rather than avoidance, to be a predictor of parenting stress among a sample of at-risk low SES mothers. Such contrasting findings may be due to the variety of samples and measures used (Jones et al., 2015). We can consider adoptive mothers and fathers an at-risk population, due to the many challenges that adoptive parents have to face when adopting a child (Viana and Welsh, 2010). In this perspective, our findings confirm the central role of anxiety in predicting parenting stress in a sample of adoptive mothers (Green et al., 2007). Previous studies show how attachment anxiety is linked to greater feelings of incompetence in parenting and to more social isolation, since anxiously-attached individuals seek, but cannot benefit from, intimate social support (Collins and Feeney, 2000; Moreira et al., 2003).

As regards fathers, their role has been less extensively studied in the literature (Jones et al., 2015). Our results support the idea that avoidance, but not anxiety, predicts parenting stress among adoptive fathers. Past research found that avoidant parents feel more distant and less involved and supportive of their children, express less desire to become a parent and lack experience with children (Rholes et al., 2006). Overall, as previously stated, in our sample adoptive fathers were more avoidant than mothers, and therefore sought and provided less support, being less involved in their relationships. Moreover, women’s motivation to adopt is generally greater than men’s, and, especially for fathers, partnership in the adoptive process plays a fundamental role for their emotional and functional involvement with the child, and this aspect is further enhanced in international adoption (Levy-Shiff et al., 1997). This can also explain why dyadic functioning did not directly predict parenting stress among fathers, but only indirectly through the influence on personal aspects such as feelings of avoidance, leading to a stronger involvement with the child and to a decrease in parenting stress (Rholes et al., 2006). On the other hand, among mothers a better dyadic relationship reduced parenting stress not only indirectly by decreasing levels of anxiety linked to personal feelings of incompetence and social isolation, but also directly, showing that perceived quality of the marital relationship is paramount for adoptive mothers, both to contain their personal feelings of anxiety and incompetence, and to support them in their parental role as mothers (Viana and Welsh, 2010).

To sum up our results as a “clinical vignette,” adoptive couples in our sample appear to be overall well-adjusted and satisfied. Husbands and wives show a positive perception of their marital and reciprocal caring relationship, reporting on average a quite similar and non-clinical level of parenting stress. The characteristics of the adoption process couples have to face in Italy may explain—at least in part—these similar perceptions. In fact, the process is very long (almost two and a half years) and comprises many reiterated psychological and economical assessments. However, mothers and fathers in our sample also provide a rather different perspective on their individual, dyadic, and parental functioning. Specifically, more women report feelings of anxiety in their marital attachment relationship, higher feeling of parental incompetence emerges. From a clinical perspective, increased feelings of anxiety could lead mothers to seek greater reassurance and approval from their partners but at the same time, due to such feelings, they may continue to question their relationship and their personal value both as wives and as mothers. Hence, their personal abilities in parenting and marital functioning are impaired, resulting in a direct increase in their levels of parenting stress. On the other hand, more men report avoidant feelings in the marital attachment relationship, higher sense of exclusion and distance emerges, leading to higher parenting stress. Feelings of avoidance lead individuals to disregard relationships, undermining the universal need to belong, which is crucial in adoptive families, especially at the initial stages. We could hypothesize that feelings of avoidance among fathers lead to a decrease in the sense of involvement in the family, which in turn leads to a stronger sense of exclusion that can be responsible for the increase in parenting stress experienced with their adoptive child.

### Limitations

Despite the unique contribution of our findings to extending knowledge about the factors involved in adoptive parents’ adaptation processes, especially as regards fathers, this study presents some limitations. First, the reduced sample size limits the generalizability of our findings to the whole population of Italian adoptive parents, and did not allow to test mothers and fathers simultaneously in the same statistical model. Replicating this study in larger and more homogenous samples (e.g., adoptive families with children from a specific age range, ethnicity and country of origin) may be useful for obtaining more reliable results. Second, there could have been some overlap between our measure of behavioral problems and our measure of parenting stress, since both refer to some extent to parents’ perceptions of child difficulties. Third, the lack of a comparison group of biological parents prevents us from drawing conclusions about specific and unique processes characterizing adoptive mothers and fathers. Fourth, this study relied exclusively on parental self-report measures, and a negative or positive reporting bias might result due to methodological variance and respondents’ personal characteristics. Despite the choice of well-established and STANDARDIZED measures, future research could benefit from a multi-method approach to increase the validity of results. Finally yet importantly, the cross-sectional nature of our study prevents us from drawing conclusions about causality. Longitudinal studies are needed to sketch the developmental trajectories of dyadic adjustment, adult and parenting stress in adoptive parents. Future research should address these issues, which need to be held in consideration when interpreting our results.

### Final Considerations and Implications for Practice

The present findings, together with prior research, document the overall protective role of dyadic adjustment and adult attachment dimensions on parenting stress in adoptive families (Lionetti et al., 2015). To the best of our knowledge, this is the first study to examine relationship among dyadic adjustment, attachment dimensions and parenting stress in a sample of adoptive mothers and fathers, highlighting the differential role of attachment avoidance and anxiety in mediating the relationship between dyadic adjustment and parenting stress as a function of parental gender. Understanding the processes through which dyadic functioning influences parenting stress holds significant implications for professionals who work with adoptive parents; interventions such as analyzing the family’s adjustment, the changes in marital relationship to adoption emphasizing the role of individual and dyadic variables involved in childrearing, could help parents -in particular in post adoption phase- to better cope reciprocally and with the child, increasing positively the newborn parental relationship, decreasing parenting stress. Moreover, intervention such as collaborative assessment (Finn, 2007) based on sharing diagnosis and assessment data and making sense of a problem together (Finn, 2007), or video feedback intervention (Alink et al., 2006) will be indicated to improve the effectiveness of positive parenting and family cooperation.

Our findings, such as ones from Lionetti et al. (2015), suggest differential protective effects of dyadic adjustment and attachment dimensions on mothers’ and fathers’ parenting stress, highlighting the importance of including both parents in adoption research. Results show that dyadic adjustment is important to directly reduce parenting stress, especially among adoptive mothers, whereas marital satisfaction has a more indirect effect on parenting stress among fathers. These findings confirm the importance of assessing and supporting marital adjustment pre- and post-adoption as an important variable for identifying couples who are suitable as prospective adoptive parents and as a resource in the post-adoption phase. More specifically, attachment dimensions represent an important pathway by which dyadic functioning has its effects on parenting stress, although differentially for mothers and fathers. It may be important for adoption professionals to recognize the importance of specific interventions aimed at reducing feelings of relationship anxiety and avoidance by building on parents’ successful marital functioning and supporting parental sense of competence and involvement for mothers and fathers, respectively. Preventive and support interventions with adoptive families, in pre- and post-adoption phases, may enable parents to increase their levels of security and involvement in the marital relationship and to strengthen individual and family resources. Hence, this would provide adoptive parents with some protective factors able to contain and modulate parenting stress, thereby enabling them to cope with the challenges stemming from adoption.

### Conflict of Interest Statement

The authors declare that the research was conducted in the absence of any commercial or financial relationships that could be construed as a potential conflict of interest.

## Footnotes

1 We re-tested the presence of a direct link between dyadic adjustment and parenting stress in the final model but it remained non-significant, confirming our final model as the most plausible for the observed data.
